# An Overview of the Role of Adipokines in Cardiometabolic Diseases

**DOI:** 10.3390/molecules25215218

**Published:** 2020-11-09

**Authors:** Tahereh Farkhondeh, Silvia Llorens, Ali Mohammad Pourbagher-Shahri, Milad Ashrafizadeh, Marjan Talebi, Mehdi Shakibaei, Saeed Samarghandian

**Affiliations:** 1 Medical Toxicology and Drug Abuse Research Center (MTDRC), Birjand University of Medical Sciences, Birjand 9717853577, Iran; farkhondeh2324@gmail.com; 2 Faculty of Pharmacy, Birjand University of Medical Sciences, Birjand 9717853577, Iran; ali.pourbagher.shahri@gmail.com; 3 Department of Medical Sciences, Faculty of Medicine of Albacete, Centro Regional de Investigaciones Biomédicas (CRIB), University of Castilla-La Mancha, 02008 Albacete, Spain; silvia.llorens@uclm.es; 4 Faculty of Engineering and Natural Sciences, Sabanci University, Orta Mahalle, Üniversite Caddesi No. 27, Orhanlı, Tuzla, Istanbul 34956, Turkey; dvm.milad73@yahoo.com; 5 Sabanci University Nanotechnology Research and Application Center (SUNUM), Tuzla, Istanbul 34956, Turkey; 6 Department of Pharmacognosy, School of Pharmacy, Shahid Beheshti University of Medical Sciences, Tehran 1996835113, Iran; talebi.m@sbmu.ac.ir; 7 Musculoskeletal Research Group and Tumour Biology, Chair of Vegetative Anatomy, Institute of Anatomy, Faculty of Medicine, Ludwig-Maximilian-University Munich, Pettenkoferstrasse 11, D-80336 Munich, Germany; 8 Noncommunicable Diseases Research Center, Neyshabur University of Medical Sciences, Neyshabur 9318614139, Iran

**Keywords:** obesity, cardiovascular disease, metabolic diseases, adipokines

## Abstract

Obesity as an independent risk factor for cardiovascular diseases (CVDs) leads to an increase in morbidity, mortality, and a shortening of life span. The changes in heart structure and function as well as metabolic profile are caused by obese people, including those free of metabolic disorders. Obesity alters heart function structure and affects lipid and glucose metabolism, blood pressure, and increase inflammatory cytokines. Adipokines, specific cytokines of adipocytes, are involved in the progression of obesity and the associated co-morbidities. In the current study, we review the scientific evidence on the effects of obesity on CVDs, focusing on the changes in adipokines. Several adipokines have anti-inflammatory and cardioprotective effects comprising omentin, apelin, adiponectin, and secreted frizzled-related protein (Sfrp-5). Other adipokines have pro-inflammatory impacts on the cardiovascular system and obesity including leptin, tumor necrosis factor (TNF), retinol-binding protein4 (RBP-4), visfatin, resistin, and osteopontin. We found that obesity is associated with multiple CVDs, but can only occur in unhealthy metabolic patients. However, more studies should be designed to clarify the association between obesity, adipokine changes, and the occurrence of CVDs.

## 1. Introduction

Obesity is already a global pandemic problem, and thus a significant threat to public health. Its worldwide incidence has doubled in recent years. Obesity has become one of the leading causes of death due to numerous co-morbidities [1,2]. These obesity-related diseases include cardiovascular diseases (CVDs), type 2 diabetes (DM2), dyslipidemia, and hypertension [3].

Adipose tissue, commonly referred to as ‘fat’, is a type of loose connective tissue that consists of two components, adipocytes and stromal vascular fraction (SVF). SVF consists of pre-adipocytes, immune system cells, mesenchymal cells, fibroblasts, endothelial precursor cells, smooth muscle cells, blood cells, and blood vessels [4].

CVDs are the leading cause of mortality in the obese population. The associated mechanisms and conditions of obesity, such as excessive accumulation of adipose tissue (obesity), are physiopathologically relevant as they can increase the risk of CVDs independently of other pathologies and alter the structure and function of the myocardium [5]. They can also influence other diseases’ progression and severity, such as dyslipidemia, diabetes, and hypertension [6].

Adipose tissue spreads throughout the body, though it is mainly found in visceral and subcutaneous depots [7]. Its function is essential for health; it specializes in the synthesis and storage of triglycerides in lipid droplets (lipogenesis) and the release of fatty acids into the systemic circulation (lipolysis) during periods of scarcity. Adipose tissue is not only an energy-preserving tissue but can also release numerous substances that act in a paracrine, autocrine, endocrine, and vasocrine way to maintain metabolic homeostasis. These substances include immunomodulatory proteins, collectively known as ‘adipokines’ or ‘adipocytokines’ [8]. The change in the expression of adipokines is probably the cause of chronic low-grade inflammation in obesity. Effects of adipokines on the inflammatory system have been reported in clinical and experimental studies, and this could be a factor influencing the pathogenesis of obesity-associated diseases such as CVD [9]. Metabolic disorders in different adipose tissue depots contribute to the fact that the profile of secretory adipokines varies between individuals, although in obesity the production of pro-inflammatory adipokines is generally favored despite their depot location [10,11]. The pro-inflammatory adipokines (i.e., TNF-α, Leptin, resistin, retinol-4 transporter protein (RBP4), lipocalin 2, angiopoietin-related protein 2 (ANGPTL2), interleukins (IL-6, IL-18), among others) outweigh the anti-inflammatory mediator (i.e., adiponectin). This pathogenic adipokines profile has been reported to promote cardiometabolic syndrome and CVDs in obesity [9,12,13].

Thus, understanding the links between obesity, cardiometabolic syndrome and CVDs, and the altered adipokines secretion profile is of great importance to developing new therapeutic strategies to treat obesity-related complications. This review collects scientific evidence from the last 5 years to discuss the triangle of obesity, cardiovascular disease, and cardiometabolic disorders, as well as several adipokines involved.

## 2. Obesity, Metabolic Health, and Cardiovascular Diseases

Metabolic syndrome (MS) or syndrome X is also known as a cardiometabolic syndrome. MS is a cluster of metabolic and cardiovascular symptoms strongly associated with DM2 and is very frequently associated with hypertension, dyslipidemia, atherosclerosis, and especially obesity. Furthermore, MS is defined by the presence of hyperglycemia (fasting plasma glucose levels ≥5.6 mmol/L), central obesity (waist circumference ≥90 cm for men, and ≥80 cm for women), low high-density lipoproteins (HDL) level (<1.03 mmol/L for men, <1.29 mmol/L for women), high total triglyceride levels (≥1.7 mmol/L), and elevated blood pressure (≥130/85 mmHg), highlighting that MS is a strong risk factor for the development of CVDs and DM2 [14].

Some evidence shows a complex relationship network between DM2, obesity, and CVDs. Thus, overweight/obesity is a risk factor for CVDs in DM2 individuals, and indeed individuals with DM2 and CVDs have a high probability of being overweight. Furthermore, obesity in DM2 patients increases the severity of metabolic disorders, which may further increase the risk of CVDs [15]. Animal models of obesity have shown metabolic disorders, including hyperlipidemia and hepatic dysfunction [16]. Indeed, a study in Bangladesh’s population showed that both overweight and obese individuals had increased serum insulin, triglyceride, homocysteine, insulin resistance, and atherogenic markers compared to normal-weight individuals [17].

Substantial weight gain is harmful to metabolic health and is also a risk factor for developing CVDs, even in young individuals. Moreover, overweight and obesity in childhood can increase the risk of cardiometabolic diseases. Overweight and obese children in free-living conditions are hyperglycemic [18]. The presence of insulin resistance in these children also aggravated the already established hyperglycemia, making them more susceptible to development CVDs and DM2 [18]. Remarkably, abnormal glucose metabolism and dyslipidemia have been observed in obese adolescents [19]. These hazardous effects can also be felt later in life, regardless of race, gender, and obesity status [20].

Insulin resistance is a central mechanism that connects all components of MS, regardless of whether they are tissue-specific or cell type-specific. Also, insulin resistance contributes to the CVDs promoting atherogenesis and plaque progression via multiple mechanisms, including changes in classic risk factors of CVDs and downregulation of insulin signaling pathways [21]. It is still unclear whether insulin resistance in obese individuals directly contributes to this by altering cardiac morphology and ventricular function. To date, studies in obese individuals have not shown a significant relationship between insulin resistance and left ventricular mass and index [22]. More future research is needed to clarify the mechanism behind morphological and mechanical alterations of the heart in obesity.

The state of metabolic health can be affected by the hormonal disorder, so the hormonal differences between the sexes determine their respective metabolic health. Alterations in hormone levels impair metabolic health, but to varying degrees depending on gender, leading to different phenotypic and metabolic characteristics in gender-specific obesity. For example, overweight/obese men have an increased lean mass, resting metabolic rate, and serum triglycerides levels, whereas women have an increased ratio of fat in body composition, fat mass, HDL-C, and leptin [23].

Also, anthropometric measurements are used to assess obesity and the primary assessment starts with the body mass index (BMI) as a marker of obesity. Other anthropometric indices are required to describe the distribution of obesity, such as waist circumference, skinfold, arm circumference, waist-to-hip ratio, and the waist-to-stature ratio, among others [24]. Also, these indices predict CVDs risk. One study showed that arm circumference values in adolescents were associated with the elevation of fasting insulin in men. In women, body mass values were associated with increased insulin and inflammation scores [19].

## 3. Obesity and Cardiovascular Diseases

The best known and most modifiable risk factors for CVDs include elevated blood pressure, hypercholesterolemia, diabetes, sedentary lifestyle, obesity, inappropriate diet, and smoking [25,26]. Moreover, experimental studies have shown an association between obesity with systemic hypertension and left ventricular hypertrophy (LVH) [16]. Of particular interest are studies showing that there is a close relationship between body mass and CVDs as even a slight increase in BMI significantly raise the risk of CVDs in the later years [27,28], and it has been observed that obese people with a higher risk of CVDs have a higher body fat percentage [29].

Also, obesity has significantly affected cardiac morphology and ventricular function [30,31]. It has also been shown that increased cardiac output and hypertension have been suggested as the mechanisms responsible for the development of LVH, decreased systolic function, and disturbed relaxation [32]. These changes usually occur over a long time and are regardless of the degree of obesity [9,33]. Interestingly, experimental studies have shown a positive correlation between epicardial and visceral fat mass with diastolic dysfunction [34]. It was further observed that following the development of obesity, the heart’s left ventricular function is impaired in animal models [35]. However, clinical investigations have not found an association between obesity and altered cardiac morphology and ventricular function [22].

Heart failure is higher in severely obese people [6,36], but once heart failure is established, a phenomenon called the ‘obesity paradox’ might be observed. The obesity paradox exhibits as obese and overweight patients with heart failure have a better prognosis than normal or underweight patients [37]. However, individuals with different degrees of obesity have marked differences in their prognosis of heart failure. Besides, the non-linear relation between the increasing degree of obesity and cardiovascular outcome of chronic heart failure has been shown [38]. A recent study showed that in acute heart failure patients the best short-term prognosis was seen in severely obese patients (BMI around 40 kg/m^2^) and the worst prognosis in the normal weight patients [39].

Obesity is also associated with atrial fibrillation. Indeed this arrhythmia is one of the most common arrhythmias in obese people [40]. A report based on the Framingham study showed that after adjustment for CVDs risk factors and the occurrence of interim myocardial infarction or heart failure, when the BMI increased by 1 unit, the atrial fibrillation increased by 4%. Also, the presence of obesity, regardless of its severity, increased the risk of atrial fibrillation by 50% [41].

It is known that different types of adipose tissue contribute differently to the effects of obesity. Depending on their location, adipose tissues consists of (A) subcutaneous adipose tissue (under the skin and stores ~80% of total body fat), and (B) intra-abdominal adipose tissues (~20% of total body fat) which consists of two parts: visceral adipose tissue (around the digestive organs) and retroperitoneal depot (around the kidney) [7]. These adipose tissue depots are remarkable for their enormous physiopathological relevance. The visceral adipose tissue is positively correlated with cardiovascular and cardiometabolic risk factors, regardless of age and gender [42]. Conversely, it has been observed that large depots of subcutaneous lower-body adipose tissue have protective effects on cardiometabolic health. However, metabolic disorders in these depots contribute to the development of obesity and its co-morbidities [43].

It should be noted that the metabolic profile is not necessarily always correlated with the excess of body fat. Given the different distribution of CVDs in different spectrums of BMI, some phenotypes have been described for obesity: (1) metabolically healthy overweight/obese individuals who are somehow resistant or protected towards cardiovascular morbidity; and (2) metabolically unhealthy individuals who are either normal-weight or overweight/obese [44]. How various phenotypes of obesity affect the risk of CVDs are not fully understood. A study in white European men showed that metabolically healthy overweight/obese individuals had no increased risk of mortality compared to metabolically healthy normal-weight individuals despite insulin resistance and sub-clinical inflammation in a 20-year follow-up. However, insulin resistance was more common in overweight or obese individuals, even if they were metabolically healthy [45].

In addition, there are biochemical and anthropometric differences between metabolically unhealthy and metabolically healthy individuals with obese/overweight, including having lower indices of BMI, waist circumference, percentage of fat mass, blood glucose, triglycerides, and insulin levels, and higher HDL-C levels in the latter group [29].

It is assumed that metabolically healthy and obese/overweight, in short, are benign [46,47]. Accordingly, one study found no increase in the risk of CVDs in metabolically healthy obese/overweight phenotypes over 12 years [48]. In contrast, in a prospective study, metabolically healthy obese people in the Greek population developed an unhealthy metabolic status during the 10-year follow-up [49]. One possible way for the delayed rise of CVDs risk in these individuals is the accumulated effects of obesity on metabolic health over time.

There are few data on the resistance of metabolically healthy, overweight/obese individuals to cardiometabolic diseases. The fact that these patients have a high risk of developing cardiometabolic and CVDs underline the need for scheduled screening of these groups through appropriate modalities.

## 4. Adipokines: Function and Mechanism

Adipose tissue comprises one of the most diverse types of cells such as adipocytes, endothelial cells, mast cells, fibroblasts, various immune cells, stem cells, etc. Interestingly, more than 600 different types of adipokines are secreted from this tissue. Adipokines consist of hormones, cytokines, growth factors, vasodilators, and several other substances with a variety of functions including important signal molecules [50,51]. The most investigated adipokines are adiponectin, leptin, resistin, chemotactic protein 1 (MCP-1), TNF-α, IL-6, IL-1β, IL-10, and transforming growth factor (TGF)-β. Furthermore, the functions and molecular mechanisms behind the adipokines’ effects are not fully clarified. Adipokines are involved in a variety of functions and can influence many different processes including modulation of energy and appetite, lipid and glucose metabolism, insulin function, endothelial cell function, inflammation, blood pressure, hemostasis, atherosclerosis, metabolic syndrome, etc. [52].

## 5. Leptin

Leptin is a peptide hormone produced and secreted by mature adipocytes from white adipose tissue including the subcutaneous adipose tissue. It consists of 167 amino acids and is encoded on chromosome 7 at the gene locus 128.24–128.26. Leptin can pass the blood-brain barrier and exerts its effect mainly in the area of the hypothalamus and is associated with the expansion of the total fatty tissue of the body. It has been reported that females have higher rates of leptin synthesis compared to males [53]. Leptin affects insulin regulation as its high levels reduce insulin secretion and its low levels stimulate insulin synthesis. Besides, leptin regulates lipid metabolism, hematopoiesis, and pancreatic β-cell function [54,55]. Leptin can affect peripheral adiposity and the central nervous system (CNS) to modulate cardiometabolic conditions. Leptin receptor-expressing cells and leptin receptor-mediated neural networks regulate the neuroendocrine output and sympathetic nervous function, leading to homeostasis of cardiometabolic condition and disruption of CNS leptin signaling causes metabolic disorders including obesity, type 2 diabetes, and hypertension [56].

Leptin controls food intake by binding to its receptor (LEPR) in the hypothalamus [56]. This leads to a reduced feeling of hunger or increased satiety [57]. It is in antagonistic redundancy to ghrelin a gastrointestinal hormone involved in the control of hunger and satiety [57]. Also, leptin appears to play a role in inflammatory processes and embryonic implantation. It has been reported that leptin increases pro-inflammatory cytokine expression in macrophages and T-lymphocytes, and stimulates inflammatory pathways such as JAK-STAT3, mitogen-activated protein kinases (MAPKs), and phosphatidylinositol-4,5-bisphosphate 3-kinase (PI3Ks). In addition, obesity is accompanied by leptin resistance (‘hyperleptinemia’) which leads to the activation of the immune cells [58]. Hyperleptinemia is associated with adipocyte dysfunction and ectopic depots in peripheral tissues and consequently insulin resistance. Individuals with leptin resistance consume more food and gain more weight [57]. Finally, it is generally accepted that leptin acts as a proinflammatory adipokine.

## 6. Adiponectin

Another adipokine, which is mainly produced by the subcutaneous adipose tissue, is adiponectin with 244 amino acids and a molecular weight of 28 kDa; the APMI gene encodes it on chromosome 3q27 [59]. In contrast to leptin, the serum adiponectin levels are lower in obese individuals. Adiponectin exhibits a wide variety of effects, including insulin sensitivity increased; fatty acid oxidation in the adipose tissue; reducing glucose release from the liver; and raising glucose uptake and adipogenesis as well as glucose metabolism and free fatty acids oxidation in the skeletal muscles [60,61].

Effects of adiponectin are facilitated through its receptors called AdipoR1 and AdipoR2. Both are found in the adipose tissue and liver; only the AdipoR1 is also found in the skeletal muscle [62,63]. The AdipoR1 contributes to adiponectin’s metabolic activity by increasing the adenosine monophosphate kinase (AMPK) activity [64,65,66]. Adiponectin can exhibit an anti-diabetic function via the AdipoR2, e.g., its binding causes an increase in insulin sensitivity, which implies the peroxisome proliferator-activated receptor (PPAR)-α activation in the liver through of this receptor [65].

The serum concentration of adiponectin is reduced in obese subjects and patients with cardiometabolic disorders [64]. Adiponectin levels are elevated with weight loss and anti-diabetic drugs. Its secretion is decreased by inflammatory mediators, proposing that inflammation may be the main factor involving in the reduction of adiponektin levels in insulin-resistant and obese conditions [65].

It has been reported that this adipokine can also prevent atherosclerosis by inhibiting the migration of monocytes/macrophages to the vascular wall and preventing the formation of foam cells. It should be emphasized that adiponectin has selective anti-inflammatory effects, i.e., it reduces endothelial cell-induced inflammation by reducing the activation of the pro-inflammatory transcription factor NF-κB (nuclear factor kappa-light-chain-enhancer of activated B-cells) [67].

Overall, adiponectin possesses anti-inflammatory, anti-diabetic, and anti-atherogenic properties. As expected, a negative correlation between obesity and adiponectin has been observed [68].

## 7. Resistin

Resistin is a polypeptide with a molecular weight of 11.3 kDa and 108 amino acids, including 11 cysteines, produced and released by adipocytes in rodents and mainly by macrophages, monocytes, and pre-adipocytes in humans [69]. The mouse resistin gene is located on chromosome 9, though the human resistin gene (Retn) is situated on chromosome 19. Mouse and human resistin share 46.7% genomic DNA homology, 64.4% mRNA sequence resemblance, and 59% amino acids identity [70]. Resistin influences insulin homeostasis, but the relationship between its serum levels and DM2, insulin resistance, or obesity is unclear and an increase in resistin levels correlated with these pathologies is still questionable [71,72]. The suggested mechanism by which the resistin affects obesity and insulin homeostasis acts on target cells both via paracrine and endocrine signaling pathways and via its receptors [73].

Resistin has also been reported to cause insulin resistance and inflammatory response. Animal models have shown that resistin can inhibit AMPK in the liver and skeletal muscle that inhibits the insulin-signaling pathway, and it has been observed that resistin can bind to the toll-like 4 receptor in the hypothalamus and activate intracellular inflammatory responses pathways as the NFκB signaling pathway [73]. Additionally, resistin induces the activation of endothelial cells via elevating the endothelin 1 expression, the intercellular adhesion molecule (ICAM-1), and the vascular cell-adhesion molecule (VCAM-1), leading to atherosclerosis in humans [74]. Measuring the resistin in metabolically unhealthy and healthy obese individuals has shown controversial results. Resistin is shown to be an inflammatory marker in the case of atherosclerosis [75]. Resistin concentrations were found to be higher in metabolically unhealthy obese individuals compared to the metabolically healthy [76], while another study found no differences [77].

## 8. Other Adipokines

Other adipokines were involved in obesity-related CVDs and cardio-metabolic disorders. The group of pro-inflammatory cytokines including TNF-α, IL-6, and IL-1β inhibit lipoprotein lipase in adipocytes, thereby increasing the lipolysis and secretion of circulating free fatty acids, leading to insulin resistance [78]. It has been reported, that one of the important anti-inflammatory adipokines in the IL-10. The IL-10 blocks pro-inflammatory cytokines such as TNF-α, IL-6, IL-1β and elevates anti-inflammatory cytokines such as IL-1Rα [79,80]. Besides, chronic inflammation in obesity leads to alterations in serum IL levels, which decreases IL-10 and increases IL-1β [81].

The transforming growth factor-β (TGF-β) is another adipokine belonging to the growth-factor family and is capable of modulating cell proliferation, differentiation, cell adhesion, migration, and death. The TGF-β levels in adipose tissue have been reported to be associated with severe obesity [82,83]. The role of TGFβ in obesity is controversial and not fully understood. While TGF-β is associated with obesity in animal models and humans, it decreases the adipogenesis process in cell culture models (3T3-F442A cells) [81].

A recently identified adipokine called S100A4 has been shown to correlate with metabolic complications of excess or dysfunctional subcutaneous white adipose tissue [84]. S100A4 is related to subcutaneous white adipose tissue and insulin resistance inflammation/adipocyte hypertrophy which is BMI-independent [85]. Moreover, S100A4 inhibits obesity, diminishes the inflammatory responses, and activates the protein kinase B (Akt) signaling [86]. Retinol-binding protein (RBP4) is another adipokine that is elevated in obesity and causes insulin resistance [87]. Vaspin and omentin are two anti-inflammatory adipokines that ameliorate insulin resistance; visfatin and lipocalin are two other pro-inflammatory adipokines that stimulate TNF-α activity [88]; and Zinc-a2-glycoprotein (ZAG) possesses desirable effects on inflammation and regulates lipid and glucose metabolism [89].

In vitro and in vivo studies demonstrated that secreted frizzled-related protein (Sfrp5) is another anti-inflammatory adipokine that participates in the pathogenesis of insulin resistance, DM2, dyslipidemia, obesity, and atherosclerotic cardiovascular disease. For the achievement of this purpose, Sfrp5 acts principally by hindering the Wnt signaling pathway [2,90,91].

Another adipokine, apelin is an endogenous peptide, well-known as a ligand of the orphan G protein-coupled receptor APJ. Apelinergic system might play role in hypertension, cardiac contractility, heart failure, DM2, and obesity. Signal transduction pathways-PI3K/Akt, extracellular signal-regulated kinase (ERK), MAPK, and endothelial NO synthase (eNOS) might be proposed as mechanisms underlying the preventive impacts of apelinergic system in CVDs [92,93].

Osteopontin is a cytokine with pro-inflammatory functions. Activation of downstream signaling pathways, comprising MAPK, ERK, c-Jun N-terminal kinases (JNK), and the PI3K/Akt pathway via osteopontin is witnessed in the protection of CVDs [94].

BATokines are released from brown adipose tissue (BAT). These adipokines protect against obesity and cardiometabolic disorders by regulating BAT function. Most of them have a role in BAT hypertrophy and hyperplasia, vascularization, and blood flow, processes that are related to BAT recruitment when thermogenic activity is increased. Batokines can affect systemic metabolism and supply the beneficial metabolic impact of BAT activation [95].

## 9. Adipokines, Obesity, and Cardiometabolic Diseases

Disorders in adipose tissue lead to a change in the secretory profile of adipokines, which is the hallmark of metabolic dysfunction. An imbalance between the formations of pro- and anti-inflammatory adipokines contribute to cardiometabolic disease and CVDs complications. Furthermore, the process that triggers the dysregulation of adipokines is complex and unknown. For example, one study shows that adipose tissue hypoxia triggers the adiponectin imbalance, and they describe an inverse relationship between blood levels of adiponectin and hemoglobin in obese men [96].

In certain conditions, such as a permanently positive energy balance, adipose tissue has a functional disorder causing several effects. Fatty acids fill-up the adipocytes and alterations in adipokines’ secretion profile, including decreased adiponectin and increased leptin levels [97]. The adiponectin/leptin imbalance increases inflammation and facilitates cholesterol accumulation, which triggers the atherosclerosis process. In this condition, low adiponectin levels prevent the repair of endothelial damage and inhibition of the inflammatory response [55].

It has also been investigated whether the change in the secretory profile of adipokines and thus metabolic health depends on the depot in which fat is stored viscerally or subcutaneously, and how this storage increases hypertrophy compared to hyperplasia. Besides, one study showed that general and visceral obesity, but not subcutaneous obesity, is related to the proinflammatory adipokine profile. It was also observed that metabolic health is more related to this adipokine profile than to total adipose tissue mass. However, since all volunteers had clinically manifest vascular disease, the results may not reflect all obese populations [98].

It is known that BMI does not reflect abdominal obesity properly. Also, abdominal obesity means an excessive accumulation of subcutaneous and visceral fat tissue. These large deposits differ in their adipogenic, lipolytic, and lipogenic capacities, and their secretory profiles (adipokines, cytokines, and other characteristic factors). It should be emphasized that visceral adipose tissue is metabolically more active than subcutaneous adipose tissue, and its dysfunction has been reported to acts as a predictor of cardiometabolic health [99]. Several anthropometric measures such as waist circumference, sagittal abdominal diameter, and waist/hip ratio are used to assess abdominal fat. It has been reported that the correlation between these three measurements of visceral fat and CVD risk factors, has been established previously [100]. Studies have also shown that higher visceral fat (measured by waist circumference or waist to hip ratio) is independently associated with lower adiponectin formation [101,102]. Interestingly, in obese individuals with DM2, the adipokines profile may differ from those without DM2. Indeed, one study showed that obese patients with DM2 had significantly higher adiponectin levels compared to non-diabetic and non-obese patients [49].

As mentioned above, the longevity of obesity is as important as obesity itself. It has been shown that the changes in adipokines secretion profile throughout the years in obesity can independently predict the CVDs in individuals with coronary artery disease and diabetes [103]. However, the association was non-linear and dependent on BMI values. It is known that the different types of obesity have different effects on the adipokines secretory profile. Thus, individuals with central obesity had increased pro-inflammatory adipokines (TNF-α, leptin) and decreased anti-inflammatory adipokines (adiponectin), compared to individuals without central obesity [104].

It has been reported that the adipokine profile, which varies by gender, can influence the complications of obesity, as well as its severity and characteristics. However, complications-related obesity may have different gender-specific mechanisms that are associated with some adipokines. As mentioned above, obese females usually have higher leptin levels. Experimental studies have shown that leptin induces hypertension and endothelial dysfunction in female mice via aldosterone-dependent mechanisms [105]. Adiponectin is known to be generally higher in females and its low levels are associated with visceral adiposity. The underlying reasons for higher adiponectin levels in females may be differences in obesity or sex hormones. However, a study in obese Australian Aboriginal women with chronic kidney disease showed that female-gender was not associated with higher adiponectin levels [102].

It is worth mentioning that the contribution of resistin to metabolic disorders is partly due to inflammation. However, it has been reported that increased serum resistin levels have been observed in overweight and obese women, which may lead to metabolic disorders, and it may be associated with minor inflammation [106].

Since metabolically healthy overweight/obese individuals are in some way resistant towards cardiometabolic complications of obesity, variations in their adipokines profile compared to metabolically unhealthy obese individuals are likely. It has been observed that metabolically unhealthy obese individuals have significantly lower leptin levels compared to metabolically healthy obese individuals [107]. However, the adiponectin did not differ between the two groups.

## 10. Adipokines, Obesity, and Cardiovascular Diseases

In general, the role of adipokines in overweight- and obesity-related CVDs is not yet fully understood. One study found that neither adiponectin nor leptin had an independent association with CVDs. However, IL-6-related signaling pathways showed a significant correlation with the occurrence of CVDs [108]. The carotid intima-media thickness (CIMT) has been reported to be a validated marker for the severity of atherosclerosis [109,110]. In obese children, the elevation of CIMT has been associated with low levels of adiponectin, higher levels of leptin, increased C-reactive proteins with high sensitivity (hsCRP), higher levels of lipid, and hypertension. However, adiponectin levels showed a negative correlation with BMI and atherogenic factors [111,112].

Different groups and blood levels of adipokines have been found depending on the obese individuals’ age. A cohort study showed that obesity was associated with higher leptin, CRP, and IL-6 levels and lower adiponectin levels from age 11 years and higher endothelial markers such as E-selectin and tissue plasminogen activator (tPA), at 15 years and onwards [113]. However, the longevity of obesity is sometimes overlooked when assessing the risk of CVDs and metabolic disorders.

**Figure 1:**
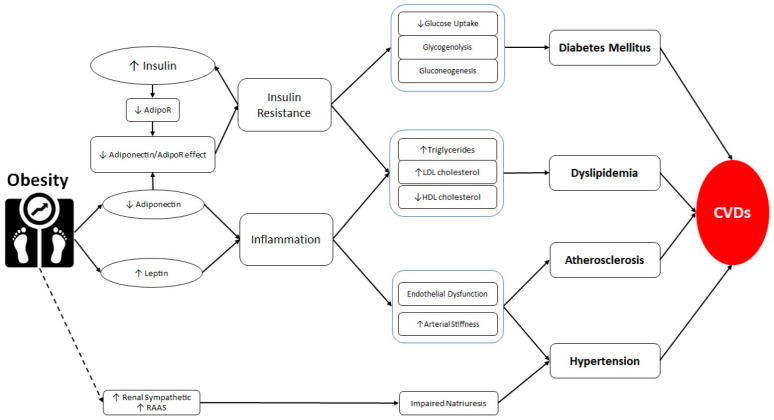
Association between obesity and cardiovascular diseases. Obesity increases leptin and decreases adiponectin. The latter induces insulin resistance via decreasing adiponectin and adiponectin receptor effect. Insulin resistance results in diabetes mellitus. Increased leptin and decreased adiponectin induces inflammation. Both insulin resistance and inflammation induce dyslipidemia. Moreover, inflammation results in atherosclerosis and hypertension through the induction of endothelial dysfunction and increased arterial stiffness. Obesity facilitates hypertension further by impairing the natriuresis balance. The complex of diabetes mellitus, dyslipidemia, atherosclerosis, and hypertension are the primary causes of cardiovascular diseases. AdipoR: adiponectin receptor; CVDs: cardiovascular diseases; RAAS: Renin-Angiotensin-Aldosterone System; AdipoR: Adiponectin Receptor; HDL: high-density lipoprotein; LDL: low-density lipoprotein.

It has been shown that fatty deposits in adolescence and adulthood are associated with higher harmful levels of adipokines and inflammatory biomarkers [113], which aggravates the atherogenic process and consequently raise the CVDs risk. When obesity complications occur, they may adversely affect some already dysregulated adipokines, which in return may worsen these obesity-related complications. One study also showed that hypertensive obese women had higher RNA expression of adiponectin than non-hypertensive women despite being anti-hypertensive [114]. Figure 1 indicates the association between “Inflammation” and “Adiponectin”/”Leptin” and heart failure.

## 11. Conclusions

It is now widely accepted that obesity affects metabolic health and increases the risk of CVDs. After increasing in adipose tissue, several changes in the anatomical structures and heart tissue function can occur even in metabolic disorders-free individuals. In addition, both the incidence and mortality rates of cardiometabolic diseases and CVDs are significantly increased in the obese population. However, controversial studies suggest that obesity does not increase the risk of CVDs in people who are free of metabolic disorders, while some studies with long follow-up periods have shown otherwise. This research area needs more comprehensive studies considering that this subgroup of obese individuals is at risk of being overlooked during medical practice.

As mentioned, adipokines and metabolites secreted by adipocytes play a central role in developing CVDs and metabolic diseases associated with obesity. Most importantly, the mechanisms involved in CVDs are attacked by the binding of adipokines to their receptors. However, the relationship of these molecules to obesity and obesity-associated diseases is still unclear. Besides, several studies reported that changes in adipokines in unhealthy metabolic patients are associated with obesity, while some studies confirmed this association in both healthy and unhealthy obese people. The elucidation of the mechanisms involved in developing inflammatory and metabolic disorders and CVDs is necessary for therapeutic approaches against the increasing epidemics of obesity and related diseases. Besides, the alterations in adipokine secretion profile in metabolically healthy and unhealthy obese individuals may be the primary tool to find protective factors against the development of obesity-related metabolic diseases and CVDs. However, given the vast number of adipokines and their different functions, further efforts and studies seem to be necessary to obtain a better picture of adipokines in obesity and obesity-related disorders.

In summary, although obesity has been identified for years as one of the significant risk factors of CVDs, unanswered scientific questions need to be addressed for this association. The discovery of sequenced genomes and biomarkers of obesity and its co-morbidities in healthy metabolic patients is an excellent strategy to prevent CVDs.

## Abbreviations

AdipoR	Adiponectin receptor
Akt	Protein kinase B
AMPK	Adenosine monophosphate kinase
ANGPTL2	Angiopoietin-related protein 2
BMI	Body Mass Index
CIMT	Carotid intima-media thickness
CVDs	Cardiovascular diseases
DM2	Type 2 diabetes
eNOS	Endothelial nitric oxide synthase
ERK	Extracellular signal-regulated kinase
HDL-C	High-density lipoprotein-cholesterol
hsCRP	high sensitivity-C-reactive proteins
ICAM-1	Intercellular adhesion molecule-1
IL	Interleukin
JAK-STAT3	Janus kinase (JAK)-signal transducer and activator of transcription (STAT)
JNK	c-Jun N-terminal kinases
LEPR	Leptin controls food intake by binding to its receptor
LVH	Left ventricular hypertrophy
MAPKs	Mitogen-activated protein kinases
MCP-1	Chemotactic protein 1
MS	Metabolic syndrome
NF-kB	Nuclear factor kappa-light-chain-enhancer of activated B-cells
PI3Ks	Phosphatidylinositol-4,5-bisphosphate 3-kinase ()
PPAR	Peroxisome proliferator-activated receptor
RBP4	Retinol-4 transporter protein
RBP4	Retinol-binding protein 4
Sfrp5	Secreted frizzled-related protein
SVF	Stromal vascular fraction
TGF-β	Transforming growth factor-β
TNF-α	Tumor necrosis factor-alpha
t-PA	Tissue-plasminogen activator
VCAM-1	Vascular cell-adhesion molecule-1
ZAG	Zinc-a2-glycoprotein
